# Development and validation of a set of patient reported outcome measures to assess effectiveness of asthma prophylaxis

**DOI:** 10.1186/s12890-021-01665-6

**Published:** 2021-09-17

**Authors:** Yalini Guruparan, Thiyahiny S. Navaratinaraja, Gowry Selvaratnam, Nalika Gunawardena, Shalini Sri Ranganathan

**Affiliations:** 1 Department of Pharmacology, Faculty of Medicine, University of Jaffna, Jaffna, Sri Lanka; 2 Department of Medicine, Faculty of Medicine, University of Jaffna, Jaffna, Sri Lanka; 3 World Health Organization, Country Office, Colombo, Sri Lanka; 4 Department of Pharmacology, Faculty of Medicine, University of Colombo, Colombo, Sri Lanka

**Keywords:** Patient reported outcome measure, Effectiveness, Asthma control, Inhaled medications, Development, Validation

## Abstract

**Background:**

In the local setting, asthma control is assessed by symptoms and signs elicited by clinicians because of the limited availability of spirometry. Hence, we intended to develop a patient reported outcome measure (PROM) with more holistic interpretation that could also serve as a tool to measure the asthma control in resource limited settings. Therefore, this study was carried out in Northern Sri Lanka to develop and validate the Asthma Control PROM (AC-PROM) Tamil to measure the effectiveness of asthma prophylaxis based on symptoms, exacerbation and limitation of activity which could also serve as an easy measure of asthma control to the provider.

**Methods:**

The AC-PROM Tamil was developed in 3 steps: item generation, item reduction and psychometric evaluation. Items were generated through thematic analysis from focus group discussions among patients with asthma. Items were converted to an interviewer administered questionnaire in Tamil in the format of 5-point Likert scale. Item reduction was done by two rounds of online Delphi surveys among 10 experts and an exploratory factor analysis among 200 patients with asthma. The face and content validity were assessed by a panel of experts during Delphi survey and patients during the pre-test of the tool. Criterion validity of the tool was assessed against the forced expiratory volume in one second of 187 patients with asthma. The cut-off value to assess the asthma control was determined by receiver operating characteristic curve. Reliability was verified by Cronbach’s alpha coefficient.

**Results:**

From thematic analysis of focus group discussions 10 items were generated. One item was removed during Delphi survey. Exploratory factor analysis indicated removal of another item with 8 items categorised into two factors. Cronbach’s alpha coefficient of factors 1 and 2 were 0.821 and 0.903 respectively, indicating good reliability. Observations made by experts and responses made by patients were incorporated to improve the clarity and relevance of the items. Criterion validity was demonstrated by significant correlation between the AC-PROM Tamil and forced expiratory volume in one second (*r* = 0.66, *p* = 0.001). The cut-off value of the AC-PROM Tamil to detect asthma control was 28.5 with 79% (95% CI 71.3–86.9) sensitivity and 71% (95% CI 61.9–79.6) specificity. The AC-PROM Tamil showed moderate accuracy (the area under the receiver operating characteristic curve = 0.796; 95% CI 0.73–0.86). Response rate of the AC-PROM Tamil was 100% and time taken to complete was 3–4 min.

**Conclusion:**

The AC-PROM Tamil is a simple, feasible and reasonably accurate tool to assesses the effectiveness of asthma prophylaxis, particularly in resource limited settings.

**Supplementary Information:**

The online version contains supplementary material available at 10.1186/s12890-021-01665-6.

## Background

Asthma is a common chronic respiratory disease, affecting 339 million people worldwide [1] and in Sri Lanka, it accounts for 13.8% of the non-communicable diseases [2]. Global Asthma Network has identified Sri Lanka as one of the high prevalence countries [1]. The Global Initiatives for Asthma (GINA) defines asthma as “variable respiratory symptoms such as wheeze, shortness of breath, chest tightness and cough that vary over the time and intensity together with variable airflow limitation” [3].

Although asthma cannot be cured, appropriate management can control the disease and enable people to enjoy good quality of life [1]. Successful asthma control comprises minimising the risk of exacerbations, reducing the adverse effects and minimising asthma-related mortality [3]. Prophylactic inhaled medications, which are being used for more than 40 years, are the mainstay in asthma control [4].

Effectiveness of asthma prophylaxis in control of asthma is assessed by improvement in the symptoms, lung function measurements and measuring biomarkers in blood, bronchoalveolar lavage and bronchial biopsy [5].

The current trend in assessing effectiveness of treatment options for chronic diseases in routine clinical care is by using patient reported outcome measures (PROMs) which capture the patients’ subjective perceptions of their health status, effects of health care interventions, functional status and their health-related quality of life that occur as a result of treatment [6–8].

Patient reported outcome measure is defined as “any report of the status of the patient’s health condition that comes directly from the patient, without interpretation of the patient’s response by a clinician or anyone else” [9]. Incorporating the patients’ perspective into clinical management could provide more holistic interpretation and comprehensive assessment of benefits of the treatment because patient can be an invaluable source of information for monitoring disease control [7, 10].

We reviewed the literature to find an asthma PROM that could be used to assess the effectiveness of asthma prophylaxis based on symptoms, exacerbation and limitation of activity. We noticed that these PROMs did not capture the holistic approach we expected. Further, forced expiratory volume in one second (FEV1) measuring capacity in our setting is very limited and asthma control is generally assessed by symptoms and signs elicited by clinicians. Hence, we intended to develop a PROM with more holistic interpretation that could also serve as a tool to measure the asthma control in resource limited settings.

Therefore, this study was conducted in Northern Sri Lanka with the aim of developing the Asthma Control PROM (AC-PROM) Tamil to measure the effectiveness of asthma prophylaxis based on symptoms, exacerbation and limitation of activity which could also serve as an easy measure of asthma control to the provider.

## Methods

This study was conducted in the Northern Province of Sri Lanka. We followed three steps namely, item generation, item reduction and psychometric evaluation, as recommended by the Food and Drug Administration to develop and validate the PROM [9]. Approval was obtained from the Ethics Review Committee, Faculty of Medicine, University of Colombo, Sri Lanka (EC-18-108) and administrative approvals were obtained from relevant authorities. Written informed consent was obtained from all participants.

Asthma was defined as “variable respiratory symptoms such as wheeze, shortness of breath, chest tightness and cough that vary over the time and intensity together with variable airflow limitation” [3]. This definition was used whenever patients were recruited for this procedure.

### Item generation

Six focus group discussions (FGDs) were conducted with 51 adult asthmatic patients who were on inhaled medications at least for 3 months to generate the items. They were recruited from the medical clinics of Teaching Hospital, Jaffna. Patients with chronic obstructive pulmonary disease, tuberculosis and congestive cardiac failure were excluded. Purposive sampling was used to recruit participants with the aim of achieving maximum variation and sampling frame confirmed that patients with a range of age, sex and disease duration were recruited. Moderator guide was developed and FGDs were moderated by a researcher. English translation of the moderator guide is attached as Additional file 2. Focus group discussions were held separately for three distinct groups based on the educational level of the participants: (1) Grade 1–5, (2) Grade 6–11 and (3) advanced level and above. For each category 2 FGDs were conducted [11]. Number of participants per group was 6–10 [12]. All FGDs were audio recorded.

### Item reduction

Both qualitative (inquiries from experts in the relevant fields using Delphi survey) and quantitative (exploratory factor analysis) methods were used in item reduction. Two rounds of online Delphi survey were conducted with a panel of 10 experts comprising respiratory physicians, general physicians, clinical pharmacologists, general practitioners and senior medical officers working in medical units. The ten items generated from FGDs with patients were submitted to the expert panel and each member was invited to rate the item in a 5-point Likert scale from 1 ‘not at all important’ to 5 ‘very important’. Items scored above the cut-off value in round 1 were subjected for round 2.

Exploratory factor analysis (EFA) is a method recommended to reduce the number of items and to group the similar items under different categories [13]. The present study used this technique on the tool with items reduced through the expert opinion, to check on further redundancy and we were expecting to remove more items. Data for EFA were obtained from 200 adult asthmatic patients who were on inhaled medications at least for 3 months, and they were recruited from a different hospital, Base Hospital, Tellipalai. Patients with chronic obstructive pulmonary disease, tuberculosis and congestive cardiac failure were excluded. Sample size for EFA was calculated based on subject to item ratio of 5:1 [14[ and minimum sample size was calculated to be 200 [15]. Systematic random sampling was used to select participants and every other participant was selected commencing from either the first or second patient. First author interviewed all 200 participants.

### Psychometric evaluation

Reliability, face, content and criterion validity and acceptability were assessed. Reliability was assessed during the EFA phase. The face and content validity were assessed by a panel of experts during Delphi survey and patients during the pre-test of the tool. The first author administered the tool to 20 patients and conducted cognitive interviews with patients. These participants were not part of FGD/ EFA or criterion validation process.

Criterion validity of the AC-PROM Tamil was evaluated with percent predicted FEV1 which is the gold standard measurement for asthma control [16]. Sample size of 187 was determined using Buderer’s formula (sensitivity 95%, specificity 85%) [17]. Participants who were on inhaled medications at least for 3 months were consecutively recruited till reaching 187. Patients who have not participated in FGD/ EFA were recruited from the medical clinics of Teaching Hospital Jaffna. Same exclusion criteria were used. Lung function tests were done in these patients using spirometer according to American Thoracic Society and European Respiratory Society Guidelines [18, 19].

The acceptability of the AC-PROM Tamil was assessed by examining the response rate, completion rate and response time among 20 patients with asthma who were being followed up at the medical clinics of Teaching Hospital Jaffna who were not part of FGD/ EFA/ pre-test or criterion validation process.

### Data analysis

Data were computerised and analysed as per the objectives. Recordings of the FGDs were transcribed into verbatim and items were generated through thematic analysis. A clinician and a clinical pharmacologist refined the items.

For item reduction by Delphi survey, scores assigned by the ten experts for each item was compiled and the mean score was calculated. Items that scored more than 3 [20] were subjected to round 2. Items which had a mean score above 4 with 80% consensus among participants [21] were selected for EFA.

Exploratory factor analysis was carried out for the nine retained items from Delphi survey. Kaiser’s criteria (eigenvalues > 1) were used for identifying number of factors and varimax rotation was used to categorize the related items under different factors [22].

Cronbach’s alpha coefficient was used to assess the internal consistency (reliability). Cronbach’s alpha coefficient 0.7 or above suggests acceptable internal consistency [23]. Criterion validity was determined using Pearson correlation coefficient between FEV1 and scores of the AC-PROM Tamil. Receiver operating characteristics (ROC) curve plotted on sensitivity against (1-specificity) was used to determine the cut-off value of the AC-PROM Tamil for asthma control. The optimal cut-off value for asthma control was determined by closest distance from the ROC curve to the upper left corner of graph which was determined by the following formula: d^2^ = [(1 − S_N_)^2^ + (1 − Sp)^2^] in which S_N_—sensitivity, Sp—specificity [22, 24]. Accuracy of the AC-PROM Tamil was measured by area under the ROC curve.

## Results

### Item generation

Fifty-one patients with asthma participated across six FGDs and mean age of the participants was 51 years (SD ± 15.47) with the male: female ratio of 1:2.5. Numbers of participants with the educational levels between grades 1 and 5, grades 6 and 11 and advanced level and above were 14, 18 and 19 respectively. The number of participants per FGD varied from 6 to 10. Each FGD lasted for 90 min. Table 1 shows the 10 items generated through thematic analysis.

**Table 1:** List of generated items

1. My cough has reduced after using the inhaler	
2. I am able to breathe without difficulty	
3. After using the inhaler, heaviness of my chest symptom has reduced	
4. I feel less tiredness	
5. I am able to sleep well	
6. The inhaler controls my wheeze	
7. After using the inhaler, I need less frequent nebulization	
8. After using the inhaler, I need less hospital admission	
9. I am able to do household activities	
10. I am able to go to work	

### Item reduction

#### Delphi survey

In round 1, scores of all 10 items were above the cut-off value. In round 2, all except one item (while on treatment, I feel less tiredness) scored above the cut-off value and the 9 items above the cut-off value were taken for EFA.

#### Exploratory factor analysis

Mean age of the 200 participants was 57 years (SD ± 13.56) with male: female ratio of 1:4. Of the 9 items, 8 had the correlation coefficient > 0.3. The item (while on treatment, I can go to work regularly) scored < 0.3, was removed. Principal component analysis identified 2 factors with eigenvalues > 1 with similar items categorised under these 2 factors (Table 2).

**Table 2:** Rotated factor loadings of the asthma control patient reported outcome measure- Tamil items

Items	Factors	
	1	2
When I am on treatment, the frequency of nebulization	.847	.164
While on treatment, the need for hospitalization	.824	.257
When I am on treatment, I can sleep well	.734	.355
When I am on treatment, I can do my routine household activities	.742	.420
While on treatment, I can go to work regularly*	.107	.096
While on treatment, I have less wheezing	.809	.289
When I am on treatment, my cough becomes less frequent	.174	.897
When I am on treatment, I can breathe without difficulty	.241	.856
When I am on treatment, heaviness of my chest is less frequent	.249	.801

*Removed because factor loading was < 0.3

Factor 1 items were related to exacerbation / limitation if activity and factor 2 items were related to asthma symptoms which are shown in Table 3.

**Table 3:** Items selected for the asthma control patient reported outcome measure-Tamil

Factor 1 (Exacerbation/limitation of activity)	Factor 2 (Asthma symptoms)
When I am on treatment the frequency of nebulization	When I am on treatment, my cough becomes less frequent
While on treatment the need for hospitalization	When I am on treatment, I can breathe without difficulty
When I am on treatment, I can do my household activities	When I am on treatment, heaviness of my chest is less frequent
When I am on treatment, I can sleep well	While on treatment, I have less wheezing

Since asthma control in a clinical follow-up context is generally assessed considering items in both factors, we pooled all 8 items as a single scale and assessed the criterion validity. This was done by converting the retained eight items to a 5-point Likert scale with 1 indicating ‘none of the time’ and 5 indicating ‘all the time’ and the total score of the tool was subjected for criterion validity. The AC-PROM Tamil is given as an Additional file 1.

### Psychometric evaluation

Cronbach’s alpha coefficient for factor 1 was 0.821, factor 2 was 0.903 and for total scale was 0.904 indicating good reliability. Observations made by experts and responses made by patients were incorporated to improve the clarity and relevance of the items.

**Fig. 1:**
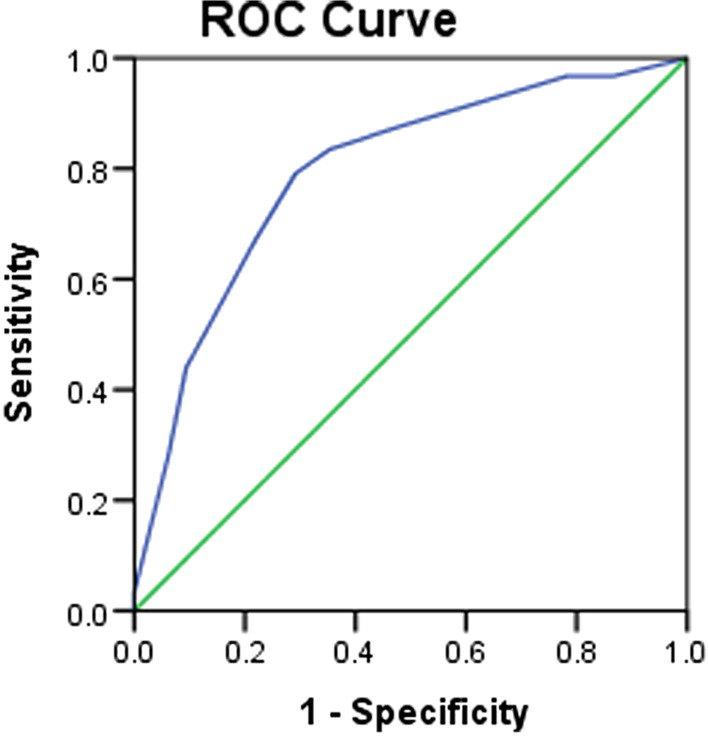
Receiver operating characteristic curve for the asthma control patient reported outcome measure Tamil

*Criterion validity*: Mean age of the 187 participants was 54.1 years (18–75 years, SD ± 12.4) and the majority (72.2%, *n* = 135) were females. The AC-PROM Tamil scores of the patients ranged from 8 to 40. Significant correlation was observed between the AC-PROM Tamil and FEV1 (*r* = 0.66, *p* = 0.001). Figure 1 shows the ROC curve for the AC-PROM Tamil. The cut-off value of the AC-PROM Tamil for asthma control was 28.5 which corresponded to the closest distance (0.11) of the ROC curve to the left-hand corner of the graph with 79.1% sensitivity and 70.8% specificity which is shown in Table 4. The area under ROC curve was 0.796 (95% CI 0.73–0.86; *p* = 0.01), indicating moderate accuracy in differentiating controlled and uncontrolled asthma.

**Table 4:** Validity of the asthma control patient reported outcome measure Tamil

Cut-off value	Sensitivity	Specificity	PPV	NPV	LR+	LR-
28.5	79.1%	70.8%	72.4%	78.6%	2.71	0.29

*PPV* positive predictive value, *NPV* negative predictive value, *LR*+ likelihood ratio positive, *LR*− likelihood ratio negative

*Acceptability of the AC-PROM Tamil:* Response rate of the AC-PROM Tamil was 100% with no missing data. Time taken to complete the AC-PROM Tamil was 3–4 min.

## Discussion

We do not have locally validated PROM to assess the effectiveness of asthma prophylaxis in Sri Lanka. Currently, asthma control is assessed by symptoms and signs elicited by clinicians using the GINA assessment of asthma control. Purpose of this study was to develop and validate a tool with more holistic interpretation to assess the asthma control employing Tamil speaking patients from Northern Sri Lanka. The AC-PROM Tamil was developed through recommended multistep methodology [9] including FGD, Delphi survey, EFA and criterion validity. As inhaled medications are the mainstay of treatment for asthma, the AC-PROM Tamil was designed to specifically assess the effectiveness of asthma prophylaxis with inhaled medications. This is the first asthma control PROM developed in Sri Lanka.

We chose FGD for item generation as it incorporates the subjective views of patients with asthma from diverse social and educational background. Item reduction was done with the aim of removing the unsuitable items and developing a simple and applicable tool. Purpose of the Delphi survey was to get reliable consensus of experts on the generated items and to identify redundant items, while EFA was carried out to further reduce the items by statistical method. During Delphi survey an item was removed as it was a vague symptom (while on treatment, I feel less tiredness) which was often used by our patients to indicate various non-sickness related events as well. During EFA another item was removed (while on treatment, I can go to work regularly) which was unsuitable for unemployed patients. Though one would normally build in more redundancy and expect removal of more items in an EFA, the fact that this was performed following a qualitative technique for item reduction in the present study can be thought as the reason for only one item to be reduced through EFA technique**.** At the end of this extensive process the retaining eight items were included in the AC-PROM Tamil. Though the tool was indicated to have two factors in EFA, we considered all items together when assessing the criterion validity as to obtain the best cut-off value to differentiate between those who are effectively treated or not. The notion that both factors were equally important in assessing asthma control and the fact that one cut-off value is practical for use in busy clinic settings was the basis for this**.** This was confirmed by the panel of clinicians. Reliability of the AC-PROM Tamil was good (Cronbach’s alpha coefficient > 0.7).

When comparing the AC-PROM Tamil with already existing PROMs for asthma, there were similarities and dissimilarities. Number of items in the other PROMs varied from 4 to 8: Asthma Therapy Assessment Questionnaire (ATAQ)—4 items; Asthma Control Test (ACT)—5 items; Asthma Control Questionnaire (ACQ)—7 items and Lara Asthma Symptom Scale (LASS)—8 items [25–28]. In ATAQ asthma control was assessed by exacerbation, limitation of activity and there were no items related to symptoms [25]. The ACT assesses the asthma control by one symptom, exacerbation (use of short acting β_2-_agonist) and limitation of activity (sleep disturbances and limitation of daily activities) [26]. Items of ACQ include two symptoms, exacerbation (use of short acting β_2_-agonist) and limitation of activity (sleep disturbances and limitation of daily activities). In addition, it requires measurement of FEV1 [27]. Out of the four PROMs we reviewed, LASS has more holistic approach as it assesses the control using four symptoms, limitation of activity by sleep disturbance and exacerbation by asthma attacks [28]. However, it did not assess the limitation of daily activities which we consider as an important indicator for asthma control from the patients’ perspective. The AC-PROM Tamil determines the asthma control based on four asthma symptoms, two items related to exacerbation (frequency of nebulization and hospitalization) and two limitations of activities (sleep disturbances and limitation of household activities). In the local setting, exacerbations of asthma are generally assessed by the frequency of nebulization and hospitalization. Like ATAQ, ACT and LASS, the AC-PROM Tamil does not require measurement of FEV1. Unlike ATAQ, ACT, ACQ and LASS, items of AC-PROM Tamil do not include self-perception of control of asthma.

Further, the AC-PROM Tamil had moderate correlation with FEV1 while ACT and LASS had mild correlation with FEV1 [26, 28]. Criterion validity of ACT was assessed against specialist assessment of asthma control and FEV1 while other three PROMs were assessed for construct validity. We have assessed the criterion validity of the AC-PROM Tamil using FEV1. Sensitivity and specificity of the cut-off value of the AC-PROM Tamil score (28.5) for asthma control were 79.1% (95% CI 71.3–86.9) and 70.8% (95% CI 61.9–79.6) respectively with the positive predictive value of 72.4% (95% CI 63.8–81) and negative predictive value of 78.6% (95% CI 70.8–86.4). Whereas sensitivity and specificity of ACT to identify uncontrolled asthma were 71.3% and 70.8% respectively with the positive predictive and negative predictive values of 72.6% and 63.3% respectively [26]. The AC-PROM Tamil has similar specificity and positive predictive values as ACT with better sensitivity and negative predicted value. The AC-PROM Tamil specifically assesses the asthma control with inhaled medications while ACT, ACQ, LASS and ATAQ assess asthma control in general.

The latest GINA assessment of asthma control comprises 4 indicators: daytime symptoms, night-time waking, short acting beta_2_-agonist use and limitation of activity [3]. The AC-PROM Tamil assesses the asthma symptoms wheeze, chest tightness, shortness of breath and cough specifically, while the GINA assessment of asthma control assesses vaguely as daytime symptoms. The AC-PROM Tamil is a numerical tool validated with FEV1while the GINA assessment of asthma control is a categorical tool. Therefore, the AC-PROM Tamil can assess the asthma control more accurately than GINA criteria. As it is a numerical tool the AC-PROM Tamil is more sensitive to change in symptom control and can be used to assess the progress. Further, the AC-PROM Tamil is a feasible tool as the response rate was 100% with no missing data and takes less than 5 min to complete.

Therefore, it could be an easy and quick measurement tool to assess the asthma control in resource limited settings. However, the actual usefulness of the AC-PROM Tamil needs to be confirmed by applying the tool in a larger population.

## Conclusions

We conclude that the AC-PROM Tamil is a simple, feasible and reasonably accurate tool that specifically assesses the effectiveness of asthma prophylaxis by determining the asthma control based on symptoms, exacerbation and limitation of activity from the patients’ perspectives. Since the AC-PROM Tamil has been validated with FEV1, it would help the practitioners to assess the effectiveness of asthma prophylaxis in health care settings where measurement of FEV1 is not feasible.

## Supplementary Information

**Additional file 1.**English translation of the asthma control patient reported outcome measure Tamil.

**Additional file 2.** English translation of the moderator guide of the focus group discussions.

## Data Availability

This is part of a larger study. The dataset generated and analysed during the current study is not publicly available but may be obtained from the corresponding author if accompanied by a reasonable request.

## Abbreviations

PROM: Patient reported outcome measure
GINA: Global Initiatives for Asthma
FGDs: Focus group discussions
EFA: Exploratory factor analysis
FEV1: Forced expiratory volume in one second
ROC curve: Receiver operating characteristic curve
ACT: Asthma control test
ACQ: Asthma control questionnaire
ATAQ: Asthma therapy assessment questionnaire
LASS: Lara asthma symptom scale
