# Supplementary material for: Development and validation of a set of patient reported outcome measures to assess effectiveness of asthma prophylaxis
Source: BMC Pulm Med. 2021 Sep 17;21:295. doi: 10.1186/s12890-021-01665-6 (PMC8449463; doi:10.1186/s12890-021-01665-6)
Supplement: Supplementary file 1 — Additional file 1.English translation of the asthma control patient reported outcome measure Tamil. [file 12890_2021_1665_MOESM1_ESM.pdf]

Additional file 1 English translation of asthma control patient reported outcome measure Tamil

1. When I am on treatment, my cough becomes less frequent

None of the time	A little of the time	Some of the time	Most of the time	All the time
1	2	3	4	5

2. When I am on treatment, I can breathe without difficulty

None of the time	A little of the time	Some of the time	Most of the time	All the time
1	2	3	4	5

3. When I am on treatment, heaviness of my chest becomes less

None of the time	A little of the time	Some of the time	Most of the time	All the time
1	2	3	4	5

4. While on treatment, I have less wheezing

None of the time	A little of the time	Some of the time	Most of the time	All the time
1	2	3	4	5

5. When I am on treatment, I can sleep well

None of the time	A little of the time	Some of the time	Most of the time	All the time
1	2	3	4	5

6. When I am on treatment, the frequency of nebulization

No reduction at all	Some extent nebulization reduced	Nebulization moderately reduced	Nebulization greatly reduced	Never required nebulization
1	2	3	4	5

7. While on treatment, the need for hospitalization

No reduction at all	Some extent hospitalization reduced	Hospitalization moderately reduced	Hospitalization greatly reduced	Never required hospitalization
1	2	3	4	5

8. When I am on treatment, I can do my routine household activities

None of the time	A little of the time	Some of the time	Most of the time	All the time
1	2	3	4	5
