# Supplementary material for: Development and validation of a set of patient reported outcome measures to assess effectiveness of asthma prophylaxis
Source: BMC Pulm Med. 2021 Sep 17;21:295. doi: 10.1186/s12890-021-01665-6 (PMC8449463; doi:10.1186/s12890-021-01665-6)
Supplement: Supplementary file 2 — Additional file 2. English translation of the moderator guide of the focus group discussions. [file 12890_2021_1665_MOESM2_ESM.pdf]

Additional file 2 English translation of the moderator guide of the focus group discussions

Could you tell me about when you first suspected that something was wrong?
Could you please describe the asthma symptoms that you have experienced?
Could you please tell me how you feel when you are taking inhaled medications?
How does asthma impact your activities?
How has asthma impacted on your emotional health or well-being?
How do you feel when you develop asthma exacerbations?
How would you minimise getting asthma exacerbations?
